# Assessing Alzheimer’s Disease Specific Barriers to Enrollment in an Observational Actigraphy Study

**DOI:** 10.1093/geroni/igaa057.904

**Published:** 2020-12-16

**Authors:** Genna Losinski, Alex Laffer, Hilary Hicks, Amber Watts

**Affiliations:** 1 University of Kansas, Lawrence, Kansas, United States; 2 The University of Kansas, Lawrence, Kansas, United States

## Abstract

There are unique challenges to recruit and enroll individuals with Alzheimer’s disease (AD) into research studies, and typical barriers to participation include the need for study partner involvement, use of invasive procedures (e.g., lumbar punctures), and lack of awareness of ongoing research. Failure to enroll this population impacts generalizability and external validity of results. The current study sought to explore reasons for non-participation in individuals with AD enrolled in the University of Kansas Alzheimer’s Disease Center (ADC) Registry. Participants were approached at their annual registry visit and asked to participate in an observational sub-study that utilized wrist-worn actigraphy to measure physical activity and sleep over a one-week period. Of the thirty-six non-participation encounters that were recorded over a 2.5 year data collection period, 28% were never recruited due to appointment cancellation, rescheduling, or no-show. Of the remaining encounters, the three most common reasons for non-participation included: physical limitations of individuals with AD (15%), unknown (28%), and study partner concerns regarding use of technology in individuals with impaired cognition due to AD (25%). Multiple study partners were concerned that the individual with AD would lose the watch, remove the watch from the wrist, or become irritated while wearing it. Findings suggest that the use of technology such as actigraphy presents an additional barrier to enrollment that is unique to individuals with AD. Future studies should consider potential interventions to address study partner concerns regarding use of technology in individuals with AD.

